# Protective Effect of Alpha-Tocopherol Isomer from Vitamin E against the H_**2**_O_**2**_ Induced Toxicity on Dental Pulp Cells

**DOI:** 10.1155/2014/895049

**Published:** 2014-01-21

**Authors:** Fernanda da Silveira Vargas, Diana Gabriela Soares, Ana Paula Dias Ribeiro, Josimeri Hebling, Carlos Alberto De Souza Costa

**Affiliations:** ^1^Oral Rehabilitation Program, Department of Dental Materials and Prosthodontics, University Estadual Paulista, UNESP, Araraquara School of Dentistry, Humaitá Street 1680, 14801-903 Araraquara, SP, Brazil; ^2^Department of Operative Dentistry, Brasilia University, School of Dentistry, Darcy Ribeiro Center, 70910-900 Brasília, DF, Brazil; ^3^Department of Orthodontics and Pediatric Dentistry, University Estadual Paulista, UNESP, Araraquara School of Dentistry, Humaitá Street 1680, 14801-903 Araraquara, SP, Brazil; ^4^Department of Physiology and Pathology, University Estadual Paulista, UNESP, Araraquara School of Dentistry, Humaitá Street 1680, 14801-903 Araraquara, SP, Brazil

## Abstract

The aim of this study was to evaluate the protective effects of different concentrations of vitamin E alpha-tocopherol (**α**-T) isomer against the toxicity of hydrogen peroxide (H_2_O_2_) on dental pulp cells. The cells (MDPC-23) were seeded in 96-well plates for 72 hours, followed by treatment with 1, 3, 5, or 10 mM **α**-T for 60 minutes. They were then exposed or not to H_2_O_2_ for 30 minutes. In positive and negative control groups, the cells were exposed to culture medium with or without H_2_O_2_ (0.018%), respectively. Cell viability was evaluated by MTT assay (Kruskal-Wallis and Mann-Whitney tests; *α* = 5%). Significant reduction of cell viability (58.5%) was observed in positive control compared with the negative control. Cells pretreated with **α**-T at 1, 3, 5, and 10 mM concentrations and exposed to H_2_O_2_ had their viability decreased by 43%, 32%, 25%, and 27.5%, respectively. These values were significantly lower than those observed in the positive control, thereby showing a protective effect of **α**-T against the H_2_O_2_ toxicity. Overall, the vitamin E **α**-T isomer protected the immortalized MDPC-23 pulp cells against the toxic effects of H_2_O_2_. The most effective cell protection was provided by 5 and 10 mM concentrations of **α**-T.

## 1. Introduction

Hydrogen peroxide (H_2_O_2_) is a thermally instable chemical agent with high oxidative power, which dissociates into free radicals and other reactive oxygen species (ROS), such as hydroxyl radicals (OH^−^), singlet oxygen (O^2−^), and superoxide anion (O_2_^−^) [1]. This molecule has been widely used in dentistry to treat discolored teeth, because of its capability to oxidize the complex organic molecules of the dental structure that respond for the darker coloration of the teeth [2]. However, these highly oxidative molecules can diffuse through mineralized tooth structures, such as enamel and dentin, to reach the subjacent pulp tissue, a specialized connective tissue responsible for maintaining the tooth viability [3, 4]. The contact of the pulp cells with ROS results in oxidative stress generation, mainly because of the imbalance between the amount of ROS and endogenous antioxidants [1]. This oxidative stress damages the cell membrane and causes cell viability reduction, extracellular matrix degradation, inflammatory tissue reaction, and even pulpal necrosis [3–5].

The treatment of dental pulp cells with antioxidants has been proposed in order to prevent the oxidative damage from components leached by dental materials and bleaching gels, which are capable of diffusing across mineralized tissues of teeth [6, 7]. Vitamin E (VE) has a recognized anti-inflammatory and antioxidant activity in different cell lineages, such as fibroblast, osteoblasts, and neurons [8]. This kind of vitamin is composed of a blend of tocopherols and tocotrienols; however, the antioxidant action of VE is mediated by the alpha-tocopherol (*α*-T) isomer [9]. The *α*-T is capable of stabilizing cell membrane against reactive oxygen species (ROS) produced during normal cellular metabolic activities, preventing the chain propagation from the oxidative stress [10]. The protective activity of this molecule against the oxidative damage related to different conditions, as atherosclerosis, diabetes, Alzheimer, and Parkinson diseases, has been widely described [8]. In view of this, it was hypothesized that the VE antioxidant property may also protect pulp cells against the oxidative toxic effects caused by components leached by dental bleaching gels. Therefore, the aim of this study was to evaluate the protective effects of different concentrations of VE *α*-T isomer against the toxicity of H_2_O_2_ applied on the immortalized odontoblast-like MDPC-23 cell line.

## 2. Materials and Methods 

The H_2_O_2_ concentration capable of reducing the cell viability by approximately 50% (IC-50) was determined. For such purpose, solutions containing decreasing H_2_O_2_ concentrations were prepared (0.035%, 0.018%, 0.009%, and 0.045%) in serum-free DMEM (Dulbecco's Modified Eagle's Medium; Sigma Aldrich Corp., St. Louis, MO, USA). Then, odontoblast-like MDPC-23 cells were seeded in DMEM supplemented with 10% fetal bovine serum (FBS; Gibco Co., Grand Island, NY, USA) and antibiotics (IU/mL penicillin, 100 *μ*g/mL streptomycin, and 2 mmol/L glutamine; Gibco Co.), in 96-well plates (1 × 10^4^ cells/well) (Costar Corp., Cambridge, MA, USA) during 72 h at 37°C and 5% CO_2_. After that, the DMEM was aspirated and 100 *μ*L of the H_2_O_2_ solutions were applied on the cells during 30 minutes. Cell viability was evaluated by the cytochemical demonstration of the succinic dehydrogenase (SDH) enzyme using the methyl tetrazolium (MTT) assay (Gibco Co.) [3, 4]. The absorbance values of the groups (570 nm) were transformed into percentages of cell viability, considering the negative control group (DMEM) as having 100% of cell viability. The 0.018% H_2_O_2_ concentration resulted in 59% of cell viability reduction and was selected to evaluate the *α*-T protective effect against H_2_O_2_ aggression.

In order to evaluate the protective effect of *α*-T against H_2_O_2_ toxicity, four decreasing concentrations of this molecule (1, 3, 5, and 10 mM) were prepared by diluting a stock *α*-T solution (Sigma Chemical Co.) in DMEM with 5% dimethyl sulfoxide (DMSO). In this way, experimental groups were formed according to the treatment of the MDPC-23 cells with different *α*-T concentrations followed by exposition or not of the cells to a 0.018% H_2_O_2_ solution for 30 minutes. To evaluate *α*-T toxicity (*α*-T+ H_2_O_2_−), the *α*-T solutions were applied on cultured cells for 60 minutes; to evaluate *α*-T protective effect against H_2_O_2_ aggression, the solutions were applied for 60 minutes and then aspirated, followed by H_2_O_2_ application for 30 minutes (*α*-T+ H_2_O_2_+). In negative control group, DMEM containing 5% DMSO was applied (*α*-T− H_2_O_2_−) on the MDPC-23 cells. In positive control group, 0.018% H_2_O_2_ was applied on the cultured cells for 30 minutes. After treatments, the MTT assay was performed and percentages of cell viability for each experimental group were determined. Data were subjected Kruskal-Wallis complemented by the Mann-Whitney test. The significance level was set at 5% and the following null hypotheses were established: (1) H_2_O_2_ does not cause toxic effects to odontoblast-like cells; (2) *α*-T cannot eliminate or at least reduce the oxidative effects of H_2_O_2_. Three independent experiments were performed at different times to demonstrate the reproducibility of data, and, in each appointment, a total of six replicates (*n* = 6) were used for each group.

## 3. Results

Table 1 shows the results for the H_2_O_2_ IC-50. The experimental groups used to assess the protective role of *α*-T against cell toxicity mediated by H_2_O_2_ are summarized in Table 2. Cell viability data obtained after cell treatment with *α*-T followed or not by exposure to H_2_O_2_ are shown in Table 3. Considering the negative control group (G1) as having 100% of cell viability, there was a 58.5% decrease in the positive control group (G2) that was lower than that observed in the experimental groups (*P* < 0.05). The cell viability reduction in groups G3, G4, G5, and G6, in which the MDPC-23 cells were treated with different concentrations of *α*-T, was 6%, 13%, 10%, and 14%, respectively. Despite being considered discrete, the cell viability reduction for G4, G5, and G6 was significant when compared with the negative control group (G1, *P* < 0.05). In groups G7, G8, G9, and G10, in which the cultured cells were pretreated with *α*-T before being exposed to H_2_O_2_, the cell viability reduction was 43%, 32%, 25%, and 27.5%, respectively. The protective effect against H_2_O_2_ cytotoxicity observed in G7, G8, G9, and G10 was significantly higher when compared to the positive control group (G2) regardless of the *α*-T concentration (*P* < 0.05). G8, G9, and G10 presented the highest values of cell viability recovery, with no significant difference among them (*P* > 0.05) (Table 3, rows). G9 and G10, which did not show significant difference when the cells were treated or not with H_2_O_2_ (*P* > 0.05) (Table 3, columns), presented the best results for cell viability recovery.

Based on the fact that H_2_O_2_ caused toxic effects to the cultured odontoblast-like cells and that *α*-T reduced the oxidative effects of this unstable chemical agent to the immortalized pulp cell line, both null hypotheses presented in this study were rejected.

## 4. Discussion

In spite of being very popular in dental offices, vital tooth bleaching has been associated with postoperative sensitivity and pulpal damage [3–5]. In view of this, different therapies have been suggested to minimize these adverse effects, including pretreatment with antioxidant agents to reduce the oxidative stress generated by bleaching gel components to the pulp cells [7]. In the present study, the biological activity of VE *α*-T isomer against the toxic effects of H_2_O_2_ to MDPC-23 cells was evaluated. This specific kind of pulp cell, which presents odontoblast phenotype, was used in this study because in mammalian teeth odontoblasts are organized in a monolayer to underlie the dentinal tissue. Therefore, odontoblasts are the first pulp cells to be reached by components of dental products capable of diffusing through enamel and dentin [11]. In addition, for over a decade, this immortalized pulp cell line has widely been used to evaluate the cytotoxicity of different dental products and their isolated chemical components [3, 4, 7].

It was shown that all concentrations of *α*-T assessed in the present study presented cell-protective effect. The MDPC-23 cells pretreated with *α*-T for 60 minutes and exposed to the H_2_O_2_ showed higher viability compared with the group exposed only to H_2_O_2_ (G2, positive control). This protective effect of *α*-T against oxidizing agents was reported in previous investigations [9, 10]. A recent study demonstrated that the combination of vitamins E and C protected brain cells against the toxic effects induced by diazinon, a widely used pesticide in agriculture that causes brain oxidative stress [12]. Another *in vivo *study found that VE plus selenium acted as a potent antioxidant agent, reducing the oxidative stress in pregnant rats and preventing the development of gestational diabetes mellitus [13]. It is known that VE is composed of a mixture of tocopherols and tocotrienols [8], which can be distinguished from each other by the lateral chain unsaturation. It has been described that *α*-T is the compound responsible for great part of the VE antioxidant action [14]. According to previous studies, *α*-T is the predominant component of biomembranes, being effective in electron donation due to the orthoposition of its methyl group, compared with the other VE isomers [15]. Therefore, *α*-T can prevent oxidative stress propagation and stabilize the cell membrane, thus preventing the disruption of the amphipathic balance of this cell structure [14]. Antioxidants such as *α*-T can stop free radicals by donating one of their electrons to the free radical. However, *α*-T does not become a new free radical because it remains stable before and after donating the electron, which characterizes its antioxidant action [14]. It has also been shown that VE can prevent diseases such as atherosclerosis as well as cardiovascular and inflammatory disorders [16]. Some researchers have reported that VE is directly involved in the maintenance of the balance of oxidative reactions generated during the inflammation [17–20]. The authors showed that this kind of vitamin can block nitric oxide synthase (iNOS), COX-2 expression, and the NF-*κ*B signaling pathway in cultured monocytes stimulated by *E. coli* LPS. Additionally, VE was capable of inhibiting the synthesis of PGE2 and inflammatory cytokines, such as TNF-a, IL-4, and IL-8. Therefore, one can consider that VE has a broad therapeutic potential. The present investigation revealed that cells exposed only to H_2_O_2_ (G2) presented a 58.5% reduction in cell viability. The toxic effect of H_2_O_2_ was also reported in previous studies in which the authors evaluated the trans-enamel and trans-dentinal cytotoxicity of high concentrations of H_2_O_2_ on odontoblast-like cells [3, 4]. On the other hand, the treatment of MDPC-23 cells with different concentrations of *α*-T prior to their exposition to H_2_O_2_ increased the cell viability by 16–33.5%.

Despite the important protective effect, *α*-T alone caused a slight cell viability reduction in those groups in which the cells were not exposed to H_2_O_2_ (G3 to G6). It was shown that 1 mM *α*-T concentration was statistically similar to the control (G1). On the other hand, 3, 5, and 10 mM *α*-T concentrations were significantly different from G1. These data suggest that an increase of the *α*-T concentration available to the cells might cause a prooxidant action of this VE isomer, resulting in reduction in the viability of the treated cells. Some studies have demonstrated the prooxidant action of *α*-T at high concentrations or in the presence of heavy metals or peroxides [21–23]. These findings could explain the results observed in those groups in which the MDPC-23 cells were exposed only to *α*-T (G3 to G6). However, while a slight prooxidant action of *α*-T was observed (6–14% cell viability reduction), this molecule was capable of minimizing the oxidant effect caused by H_2_O_2_ on cultured MDPC-23 cells (G7 to G10). The most relevant protective effects were obtained with 5 mM (G9) and 10 mM (G10) *α*-T concentrations, in which 33.5 and 31% of cell viability recovery were observed, respectively. Since no significant difference was found between G9 and G10, it may be suggested that the best *α*-T concentration for pretreatment of odontoblast-like cells would be 5 mM. This is not only because of the protective effect of this molecule against the H_2_O_2_ cell damage but also due to its slight toxicity (G5–10% cell viability reduction).

Overall, this *in vitro* study demonstrated the potential of *α*-T as an antioxidant agent because this VE isomer was capable of protecting pulp cells against the harmful effects of H_2_O_2_, which is the main active component of tooth bleaching gels. Although the present laboratory-based results cannot be directly extrapolated to clinical situation, the original data obtained under the tested experimental conditions are promising.

## 5. Conclusion

It can be concluded that previous exposition of odontoblast-like MDPC-23 pulp cells to VE *α*-T isomer protects this cell line against the toxic effects generated by hydrogen peroxide *in vitro*. These data can drive further *in vivo* studies with the purpose of establishing specific therapies capable of preventing or at least minimizing the pulpal damage caused by tooth bleaching techniques widely used in dentistry. This may avoid the postbleaching tooth sensitivity, making this esthetic clinical procedure safer and more comfortable to the patients.

## Figures and Tables

**Table 1:** Results of the viability of the MDPC-23 cells exposed to different hydrogen peroxide (H_2_O_2_) concentrations for determination of the IC-50.

H_2_O_2 _concentration	Cell viability (%)
0	100
0.035%	5
0.018%	41
0.009%	77
0.0045%	72

**Table 2:** Control and experimental groups (*n* = 6) formed according to the treatment of the MDPC-23 cells with different alpha-tocopherol (*α*-T) concentrations followed by exposure or not to hydrogen peroxide (H_2_O_2_).

Groups	Treatment
G1	(*α*-T− H_2_O_2_−)
G2	(*α*-T− H_2_O_2_+)
G3	(1 mM+ H_2_O_2_−)
G4	(3 mM+ H_2_O_2_−)
G5	(5 mM+ H_2_O_2_−)
G6	(10 mM+ H_2_O_2_−)
G7	(1 mM+ H_2_O_2_+)
G8	(3 mM+ H_2_O_2_+)
G9	(5 mM+ H_2_O_2_+)
G10	(10 mM+ H_2_O_2_+)

**Table 3:** Percentage of viability of MDPC-23 cells treated with different alpha-tocopherol concentrations followed by exposure or not to hydrogen peroxide.

H_2_O_2_	Alpha-tocopherol concentrations				
	0	1 mM	3 mM	5 mM	10 mM
0%	100.5 (97–104)^a,A,G1^	94 (87–100)^ab,A,G3^	87 (86–91)^bc,A,G4^	90 (79–92)^bc,AB,G5^	86 (77–88)^c,AB,G6^
0.018%	41.5 (36–43.5)^a,B,G2^	57 (52–60)^b,B,G7^	68 (64–73)^bc,B,G8^	75 (67–84)^c,A,G9^	72.5 (69–78)^c,A,G10^

Lowercase letters permit comparisons within rows while uppercase letters permit comparisons within columns. Groups identified with the same letters do not differ significantly (Mann-Whitney test, *P* > 0.05).

## References

[1] Cecarini V, Gee J, Fioretti E (2007). Protein oxidation and cellular homeostasis: emphasis on metabolism. *Biochimica et Biophysica Acta*.

[2] Sulieman MAM (2008). An overview of tooth-bleaching techniques: chemistry, safety and efficacy. *Periodontology 2000*.

[3] Trindade FZ, Ribeiro APD, Sacono NT (2009). Trans-enamel and trans-dentinal cytotoxic effects of a 35% H_2_O_2_ bleaching gel on cultured odontoblast cell lines after consecutive applications. *International Endodontic Journal*.

[4] Soares DG, Ribeiro AP, da Silveira Vargas F, Hebling J, de Souza Costa CA (2013). Efficacy and cytotoxicity of a bleaching gel after short application times on dental enamel. *Clinical Oral Investigations*.

[5] de Souza Costa CA, Riehl H, Kina JF, Sacono NT, Hebling J (2010). Human pulp responses to in-office tooth bleaching. *Oral Surgery, Oral Medicine, Oral Pathology, Oral Radiology and Endodontology*.

[6] Majd ES, Goldberg M, Stanislawski L (2003). In vitro effects of ascorbate and Trolox on the biocompatibility of dental restorative materials. *Biomaterials*.

[7] Lima AF, Lessa FCR, Gasparoto Mancini MN, Hebling J, de Souza Costa CA, Marchi GM (2010). Transdentinal protective role of sodium ascorbate against the cytopathic effects of H_2_O_2_ released from bleaching agents. *Oral Surgery, Oral Medicine, Oral Pathology, Oral Radiology and Endodontology*.

[8] Aggarwal BB, Sundaram C, Prasad S, Kannappan R (2010). Tocotrienols, the vitamin E of the 21st century: its potential against cancer and other chronic diseases. *Biochemical Pharmacology*.

[9] Lu N, Chen W, Peng Y (2011). Effects of glutathione, Trolox and desferrioxamine on hemoglobin-induced protein oxidative damage: anti-oxidant or pro-oxidant?. *European Journal of Pharmacology*.

[10] Upadhyay J, Misra K (2009). Towards the interaction mechanism of tocopherols and tocotrienols (vitamin E) with selected metabolizing enzymes. *Bioinformation*.

[11] Goldberg M, Smith AJ (2004). Cells and extracellular matrices of dentin and pulp: a biological basis for repair and tissue engineering. *Critical Review of Oral Biologics and Medicine*.

[12] Yilmaz N, Yilmaz M, Altuntas I (2012). Diazinon-induced brain toxicity and protection by vitamins e plus C. *Toxicology and Industrial Health*.

[13] Guney M, Erdemoglu E, Mungan T (2011). Selenium-vitamin E combination and melatonin modulates diabetes-induced blood oxidative damage and fetal outcomes in pregnant rats. *Biological Trace Element Research*.

[14] Wang X, Quinn PJ (1999). Vitamin E and its function in membranes. *Progress in Lipid Research*.

[15] Parker RS (1989). Dietary and biochemical aspects of vitamin E. *Advances in Food and Nutrition Research*.

[16] Niki E (2011). Do free radicals play causal role in atherosclerosis? Low density lipoprotein oxidation and vitamin E revisited. *Journal of Clinical Biochemistry and Nutrition*.

[17] Wintergerst ES, Maggini S, Hornig DH (2007). Contribution of selected vitamins and trace elements to immune function. *Annals of Nutrition and Metabolism*.

[18] Reiter E, Jiang Q, Christen S (2007). Anti-inflammatory properties of *α*- and *γ*-tocopherol. *Molecular Aspects of Medicine*.

[19] Jiang Q, Yin X, Lil MA, Danielson ML, Freiser H, Huang J (2008). Long-chain carboxychromanols, metabolites of vitamin E, are potent inhibitors of cyclooxygenases. *Proceedings of the National Academy of Sciences of the United States of America*.

[20] Wu D, Meydani SN (2008). Age-associated changes in immune and inflammatory responses: impact of vitamin E intervention. *Journal of Leukocyte Biology*.

[21] Bowry VW, Ingold KU, Stocker R (1992). Vitamin E in human low-density lipoprotein. When and how this antioxidant becomes a pro-oxidant. *Biochemical Journal*.

[22] Tokuda M, Takeuchi M (1995). Effects of excess doses of *α*-tocopherol on the lipids and function of rainbow trout liver. *Journal of Nutritional Science and Vitaminology*.

[23] Pearson P, Lewis SA, Britton J, Young IS, Fogarty A (2006). The pro-oxidant activity of high-dose vitamin E supplements in vivo. *BioDrugs*.

